# The "Censure" on the Metropolitan Hospital

**Published:** 1897-01-02

**Authors:** 


### THE INSTITUTIONAL WORKSHOP.

### THE "CENSURE" ON THE METROPOLITAN HOSPITAL.

Libel by jury is one of the great difficulties which hospitals which depend on voluntary contributions have to contend with at the present day. A jury, led astray by sympathy for suffering, and perhaps in some cases influenced by a general suspicion of the powers that be, even though they only appear in the guise of the officials of a charity, will sometimes return a verdict not only contrary to the truth and unjust to officers who are spending their lives for the good of the sick poor who come under their care, but cruelly unjust to the poor themselves, in that it may divert from the charities on which they depend in their hour of need those gifts by which they are supported. Such things are constantly happening. The last instance that has come before us is the case of an inquest held on December 21st respecting the death of a woman thirty-eight years old, who was taken by the police to the Metropolitan Hospital early on the preceding Friday morning, having been run over in the street. The hospital was full, so she was accommodated on a couch until the afternoon, when she was transferred to the Whitechapel Infirmary at the expense of the hospital. The following is the account of the case which appeared in the *Daily Mail*, December 22nd:—

"CENSURE ON HOSPITAL AUTHORITIES.

"Mr. Baxter held an inquiry at Whitechapel Infirmary last night respecting the death of Elizabeth Smith, thirty-eight years old, living at 58, Flower and Dean Street, Spitalfields.

"Alice Gosling stated that on Friday morning she was informed that deceased had been run over, and was lying in the Metropolitan Hospital, Kingsland Road. Witness went there and found deceased lying on a couch in a cold room complaining of great pain. She said she was very thirsty. The people at the hospital said they were going to send her to the infirmary.

"The Coroner: Do you mean to say that the hospital authorities let the woman lie there for hours?—Witness: She had been there all night, sir, and there was not a spark of fire in the grate.

"Police-sergeant Fogwell, 28 H, stated that at 1.30 on Friday morning he was called to the deceased, who had been run over near Shoreditch Church. She was so severely injured that he obtained the ambulance and conveyed her to the Metropolitan Hospital. When he reached there no notice was given him that all the beds were full, and he left the patient.

"Dr. Toye, of the Metropolitan Hospital, said that he examined the woman on her admission, and found her suffering from fractured ribs. All the beds in the hospital were full, so the deceased was kept in the surgery.

"The Coroner: And sent away in a cab, although even the policeman saw that she was too ill to be moved in a cab?

"Witness: I did not consider her so seriously injured. —A Juror: Then you ought to if you examined her. (Hear, hear.)

"The jury returned a verdict of 'Accidental death,' and added that the hospital authorities had been guilty of gross remissness, and in future they should inform the police when the hospital was full."

It is unnecessary to say how bad an effect on subscriptions such a statement must have, or how the sick poor of the district must suffer from the publication of such a report. So impressed were we with this that we immediately set on foot inquiries into the whole of the circumstances. We obtained signed statements from the house surgeon, the dresser, the nurse, the night porter, the hall porter, and an out-patient who happened to have to wait in the surgery for a considerable time that morning and saw what was going on. As a result of these inquiries we formulated the following statement, which we sent to the Editor of the paper in which we had seen the report of the case:—

To the Editor of *The Daily Mail*.

THE METROPOLITAN HOSPITAL.

Sir,—We observe in your issue of the 22nd instant an account of an inquest on Eliza Smith, in which serious reflections are made upon the management of the above institution. We have made enquiries, and find that the hospital authorities were not only not to blame, but that they exhibited unusual care in the management of the patient referred to, as the following facts will testify. Connected with the receiving room of this hospital is a smaller room called the surgery which contains a bed, so that doubtful cases may be retained for the night when, as in the present instance, the whole of the beds in the hospital are occupied. The deceased was brought to the Metropolitan Hospital at 2 a.m. on the 18th instant. She was examined and found to be suffering from fractured ribs. At the time she was suffering from combined effects of drink and shock. The patient was properly strapped and bandaged, was placed on the bed in the surgery, supported by a bed rest and covered with blankets. A large fire was burning in the room until the hot air apparatus was heated, when the fire was allowed to go out. During the whole night the patient was attended by the night-superintendent, and provided with hot milk at frequent intervals. On examining the patient in the morning, the house surgeon found her much better, the effects of the drink had passed off, she made no complaint, and seemed satisfied with the treatment she had received. An examination of the chest showed no evidence of any intra-thoracic complication such as pneumonia, and the patient in the opinion of the house surgeon being fit for removal, he gave instructions accordingly, and before the removal of the patient she was given a pint of beef-tea, some hot milk, and bread and butter.

These facts have been supplied to us by the house surgeon, who adds that the patient was seen by another member of the resident staff besides himself.

Metropolitan hospitals have many difficulties to contend with during this busy season when accidents are more frequent than usual, and we are confident you will gladly give publicity to the above facts in justice to the Metropolitan Hospital and its managers.—We are, Sir, your obedient servant,

THE EDITOR.

THE HOSPITAL, 28 & 29, Southampton Street, W.C.
December 24th, 1896.

Those who are conversant with hospital management know how ready subscriptions are to fade away at the breath of calumny, and how much the resources of a hospital may be crippled by a damaging report, even when published in undoubtedly good faith. In view, then, of the fact that the Metropolitan Hospital serves a very poor district, and that even at the present time many of its wards are closed from want of funds, we think it of the greatest importance that it should be widely known that the "censure" administered by the jury was entirely undeserved, and we can but feel regret that a newly-established newspaper which seems so full of promise should slip into the bad journalistic habit of speaking evil, even of charity, if only sensation can be produced

